# Effects of Tempeh Fermentation with *Lactobacillus plantarum* and *Rhizopus oligosporus* on Streptozotocin-Induced Type II Diabetes Mellitus in Rats

**DOI:** 10.3390/nu10091143

**Published:** 2018-08-22

**Authors:** Ying-Che Huang, Bo-Hua Wu, Yung-Lin Chu, Wen-Chang Chang, Ming-Chang Wu

**Affiliations:** 1 Graduate Institute of Bioresources, National Pingtung University of Science and Technology, Pingtung 91201, Taiwan; huangleo0811@gmail.com; 2 Department of Food Science, National Pingtung University of Science and Technology, Pingtung 91201, Taiwan; david9097@yahoo.com.tw; 3 International Master’s Degree Program in Food Science, International College, National Pingtung University of Science and Technology, Pingtung 91201, Taiwan; ylchu@mail.npust.edu.tw; 4 Department of Food Science, National Chiayi University, Chiayi 60004, Taiwan; d99641001@ntu.edu.tw

**Keywords:** tempeh, lactic acid bacteria, short chain fatty acids, metabolic syndrome, high fat diet, feces

## Abstract

The increased consumption of high fat-containing foods has been linked to the prevalence of obesity and abnormal metabolic syndromes. *Rhizopus oligosporus*, a fungus in the family Mucoraceae, is widely used as a starter for homemade tempeh. Although *R. oligosporus* can prevent the growth of other microorganisms, it grows well with lactic acid bacteria (LAB). *Lactobacillus plantarum* can produce β-glucosidase, which catalyzes the hydrolysis of glucoside isoflavones into aglycones (with greater bioavailability). Therefore, the development of a soybean-based functional food by the co-inoculation of *R. oligosporus* and *L. plantarum* is a promising approach to increase the bioactivity of tempeh. In this study, the ameliorative effect of *L. plantarum* in soy tempeh on abnormal carbohydrate metabolism in high-fat diet (HFD)-induced hyperglycemic rats was evaluated. The co-incubation of *L. plantarum* with *R. oligosporus* during soy tempeh fermentation reduced the homeostatic model assessment of insulin resistance, HbA1c, serum glucose, total cholesterol, triglyceride, free fatty acid, insulin, and low-density lipoprotein contents, and significantly increased the high-density lipoprotein content in HFD rats. It also increased the LAB counts, as well as the bile acid, cholesterol, triglyceride, and short-chain fatty acid contents in the feces of HFD rats. Our results suggested that the modulation of serum glucose and lipid levels by LAB occurs via alterations in the internal microbiota, leading to the inhibition of cholesterol synthesis and promotion of lipolysis. Tempeh, which was produced with both *L. plantarum* and *R. oligosporus*, might be a beneficial dietary supplement for individuals with abnormal carbohydrate metabolism.

## 1. Introduction

The consumption of fast food, fried food, and high-fat foods is increasing along with changes in lifestyle. Therefore, the incidence of metabolic syndrome is increasing and is expected to become a major issue worldwide. It is characterized by high blood pressure, high blood sugar, hypertriglyceridemia, obesity, and low high-density lipoprotein (HDL) levels in the blood. In addition, metabolic syndrome is associated with an increased risk of type II diabetes and cardiovascular diseases. Therefore, the WHO predicts that the prevalence of diabetes mellitus (DM) will increase to 5.92 billion individuals by 2035, and Asia is one of the regions with the highest patient population [1]. Preliminary estimates are predicted to increase to 42.3 million for patients with diabetes mellitus in Asia in 2080 from 20.8 million populations in 2000, and economic development, high-fat foods, fried food, etc., are likely to be the primary underlying causes [1].

*Lactobacillus* has wide applications in probiotics and has many advantages among humans and animals. It will be beneficial to administer active microorganisms to hosts when probiotics are supplied in sufficient quantity [2]. The study also shows that probiotics play an important role in preventing and treating chronic metabolic diseases or immune-related diseases. Many studies have shown that lactic acid bacteria (LAB) are beneficial for human health, e.g., they could decrease the total cholesterol in blood and they have favorable effects in patients with type II diabetes [2]. It remarkably increased fecal and bile acid cholesterol levels after administration of *Lactobacillus plantarum*. Furthermore, it helped decrease the total blood cholesterol levels after moderate intake of *Lactobacillus plantarum* [2,3]. Recently, numerous phytochemicals have been reported in soybeans and fermented soybean products. In particular, isoflavones genistein and daidzein are beneficial for humans and isoflavones can prevent cardiovascular diseases, cancers, metabolic syndrome, or help to treat osteoporosis because it can mimic estrogen in humans [2,4]. Furthermore, certain animal studies reported that isoflavones can either decrease body weight or increase insulin levels; moreover, it plays an important role in modulating serum glucose levels in diabetic rats [2]. Numerous complex compounds are metabolized/decomposed by microorganisms to generate compounds of higher nutritional value, such as increasing aglycone during soybean fermentation [4].

Tempeh is a fermented soybean product that originated in Indonesia. Tempeh is rich in soy protein and genistein, which have beneficial effects on the regulation of high blood sugar and prevent diabetes [5]. The processing of tempeh involves the addition of *Rhizopus* spp. to cooked, peeled soybeans for fermentation at 37 °C for five days. The weather in Indonesia is wet and hot, and accordingly, tempeh can be made at room temperature [6]. Some studies have reported that tempeh, which prevents diarrhea and anemia and is richer in vitamins and minerals than unfermented soybean, contains many vitamins B_12_ and antioxidants [4]. Furthermore, genistein, daidzein, and β-sitosterol in tempeh prevent cancers, cardiovascular diseases, type II diabetes, and blood glucose regulation [7]. Tempeh also significantly decreases phytic acid and trypsin (antinutritive factors) levels during fermentation. This is one of the reasons why tempeh is popular, especially among vegetarians, in Asia, Europe, and the Americas because of its beneficial functions [4,7,8].

Many studies have shown that fermented soybean and LAB are effective for the prevention of type II diabetes [9,10]. However, the effects of the co-fermentation of *Lactobacillus plantarum* and *Rhizopus oligosporus* on type II diabetes have not been evaluated. Therefore, we prepared tempeh while using both *L. plantarum* and *R. oligosporus* (a common fungus used as a starter for tempeh) and administered it to rat models of diabetes, with HFD-induced high serum glucose and cholesterol. The objective of this study was to develop a strategy to improve the quality of life in patients with metabolic syndrome based on alternative food therapy.

## 2. Materials and Methods 

### 2.1. Sample Preparation

Kaohsiung Number 9 soybeans were used for co-fermented tempeh. Soybeans were washed and soaked for 12 h and the outer membranes were removed. After drying, water (twice the weight of soybeans) and 1% lactic acid were added, followed by cooking at 100 °C for 30 min. Next, *L. plantarum* and *R. oligosporus* were inoculated at 30 °C in a fermentative environment for 48 h after samples were cooled. Normal tempeh was prepared according to the same procedure with only *R. oligosporus*. All of the samples were stored at −20 °C in a refrigerator until the central temperature reached −18 °C, and samples were then freeze-dried for 48 h. After the water was removed, samples were milled and stored at −20°C. In addition, normal diet (LabDiet 5001) was purchased from Young Li Trading Co., Ltd. (New Taipei, Taiwan) The composition of the HFD was normal diet: cholesterol: coconut oil = 73:2:25 [11]. 

### 2.2. Animals and Diets

Eight-week-old male Sprague–Dawley (SD) rats were obtained from BioLASCO Taiwan Co., Ltd. (Taipei, Taiwan). The animals were housed in a room with an alternate light/dark cycle (12 h), a temperature of 25 ± 2 °C, and a relative humidity of 55−60%. All rats were fed experimental diets ad libitum with free access to drinking water at all times. After two weeks of adaptive feeding, the rats were randomly assigned to groups of eight animals each and fed different experimental diets as follows: rats in the control group were fed a normal chow diet with 13.5% kcal fat (Laboratory Rodent Diet 5001; Lab Diet/PMI Nutrition International, Purina Mills LLC, Gray Summit, MO, USA) and rats in the negative control group and treatment groups were fed the HFD (coconut oil 25%, cholesterol 2%, feed powder 73%) modified, as described in Gandhi et al. [11]. Diabetes was induced by treatment with 30 mg/kg STZ and 45 mg/kg nicotinamide for four weeks. Rats were induced by 20 mg/kg STZ again if their serum glucose levels did not reach 150 mg/dL after one week of induction. Rats in the treatment groups (8 rats/group) were separated into the normal diet group (control group), negative control group (HFD, SH group), and positive control group fed pioglitazone (10 mg/kg body weight/day, SHP group) in the last four weeks. The other rats were orally administered cooked soybean (40 mg/kg body weight/day, SHS group), tempeh (40 mg/kg body weight/day, SHL group), or probiotic fermented tempeh (40 mg/kg body weight/day, SHTL group) in the last four weeks. The total study period was 14 weeks for all groups. Food intake and body weight were measured weekly for the duration of the experiment. The animals were maintained in accordance with the National Pingtung University of Science and Technology and Tajen University guidelines for the care and use of laboratory animals. The animal study protocols were approved by the Ethics Committee at the Tajen University (Approval No. 105-10).

### 2.3. Serum Samples 

All blood samples were solidified at room temperature for 30 min after collection. Centrifugation at 3000× *g* for 20 min, the supernatant was obtained and stored at −80°C before analysis.

### 2.4. Fasting Serum Glucose 

Before the fasting serum glucose test, all rats were fasted overnight (14–16 h). Blood from the tail artery was collected (0.1 mL/rat) and analyzed while using a blood-glucose meter.

### 2.5. Oral Glucose Tolerance Test (OGTT)

The OGTT assay followed a similar protocol to that of the fasting serum glucose test. All of the rats were fasted overnight (14–16 h) and weighed. Blood was then collected from the tail artery (0.1 mL/rat) and analyzed using a blood-glucose meter. All animals received 1.5 g of glucose/kg body weight. Blood was sampled from the tail vessels of conscious animals before the load (*t* = 0) and 30, 60, 90, and 120 min after glucose administration. The samples were allowed to clot for 30 min, centrifuged (3000× *g*, 20 min), and evaluated while using a blood-glucose meter. 

### 2.6. Biochemical Measurements

Commercial kits for determining the levels of free fatty acids (FFA), HbA1c, high-density-lipoprotein-cholesterol (HDL-C), insulin, and low-density-lipoprotein-cholesterol (LDL-C) in rats were obtained from Randox Laboratories (Crumlin, Co., Antrim, UK). The biochemical assays were performed according to the protocols provided by Randox Laboratories.

### 2.7. Homeostasis Model Assessment-Insulin Resistance (HOMA-IR)

The homeostasis model assessment for insulin resistance (HOMA-IR) was calculated via the following equation: fasting serum insulin (mU/L) × fasting glucose (mmol/L)/22.5 [12].

### 2.8. Stool Assay

Total LAB in stool samples were determined while using a 1.0-g stool sample diluted 10–1000 times with double distilled endotoxin-free water. Next, 1.0 mL of the sample was added to Lactic Acid Bacteria Count Plates 6461 (3M Petrifilm, St. Paul, MN, USA). Samples were analyzed after incubation for 48 h at 37 °C. For short chain fatty acid (SCFA) detection, the protocol described by Holben [13] was used, with modifications. First, 910 μL of absolute alcohol and 90 μL of pivalic acid (5 mg/mL) were added to 0.5 g of the stool sample and vortexed for 2 min. Next, 500 μL of 0.8 M perchloric acid was added and vortexed for 5 min, followed by centrifugation for 1 min at 13,000 rpm. Then, 0.5 mL of the supernatant was mixed with 50 μL of 4 M KOH for 5 min, and 250 μL of oxalic acid solution was added at 4 °C for 60 min. Finally, the sample was centrifuged for 1 min at 13000 rpm again and the supernatant was passed through a 0.22-μm filter. All of the samples were analyzed while using Mass Selective Detector 5973Network, HP-INNOWax (Capillary column: 30 m, inner diameter: 0.25 mm, particle size: 0.25 µm, detector: Mass Selective Detector 5973Network, gas: Helium, split rate: 5:1, column flow rate: 2 mL/min, total flow rate: 15 mL/min, injector temperature = 200 °C, oven temperature = 100 °C, detector temperature = 200 °C, initial temperature = 100 °C for 1 min, heating procedure of 2 °C/min until reaching 110 °C for 2 min, then 3 °C/min until reaching 170 °C for 1 min, final heating at 10 °C/min until reaching 200 °C for 2 min). Each sample (1 μL) was used for gas chromatography injection for 32 min, and then a mass spectrometer was used to compare acetic acid, propionate, and butyrate, as described previously [13]. Cholesterol, triglycerides, and cholic acid were analyzed while using ELISA kits (BioVision Inc., Milpitas, CA, USA). All tests were performed according to the protocols provided by BioVision Inc.

### 2.9. Next-Generation Sequencing Analysis of Stool Samples

#### 2.9.1. Amplicon Library Construction and Sequencing

Total bacterial DNA from 5 g of rat feces was isolated and purified using the PowerSoil^®^ DNA Isolation Kit (Mo Bio, Qiagen, Hilden, Germany). A 16S rDNA region (V3–V5 hypervariable region) from purified total bacterial DNA was amplified via PCR to produce 400-bp DNA fragments for further purification. The specific PCR primers were as follows: forward primer overhang adaptor (5′–TCGTCGGCAGCGTCAGATGTGTATAA GAGACAG–3′) and reverse primer overhang adaptor (5′–GTCTCGTGGGCTCGGAGATGTG TATAAGAGACAG–3′). Amplicons were generated while using a high-fidelity polymerase (AccuPrime; Invitrogen, Carlsbad, CA, USA), purified using a Magnetic Bead Capture Kit (Ampure; Agencourt, Beverly, MA, USA), and quantified using a fluorometric kit (QuantIT PicoGreen; Invitrogen, Carlsbad, CA, USA). PCR conditions were 30 cycles of 30 s at 95 °C, 30 s at 55 °C, and 30 s at 72 °C, and a final extension for 5 min at 72 °C. The purified amplicons were then pooled in equimolar concentrations using a SequalPrep Plate Normalization Kit (Invitrogen, Carlsbad, CA, USA). The final concentration of the library was determined using an SYBR Green Quantitative PCR (qPCR) assay and the size distribution of the library was determined using Caliper LabChip. 16S rRNA-specific regions were then sequenced using a MiSeq sequencer (Illumina, San Diego, CA, USA).

#### 2.9.2. Bioinformatic Analysis 

Raw reads from the MiSeq sequencer for the metagenomic workflow were analyzed while using QIIME (http://qiime.org/). Reference sequences in Greengenes gg_13_8 (99_otus.fasta) were used in the analysis (Greengenes database, http://greengenes.lbl.gov/). The Ribosomal Database Project (RDP) classifier (http://rdp.cme.msu.edu/classifier/) was used to classify the 16S rDNA sequences into distinct taxonomic categories that are based on sequence alignments. The operational taxonomic units (OTUs) for Lactobacillus species were determined by BLAST searches and groups were preliminarily assigned by alignments with the NCBI genome database. All 16S rDNA sequences were mapped to the RDP database while using QIIME and divided into groups corresponding to their taxonomy at the level of order and were then assigned to OTUs. A sequence similarity exceeding 0.95 was the threshold for OTUs, according to the value for species distinction in microbiology. 

### 2.10. Statistical Analysis

All results are reported as means ± SD and the differences between the control and tempeh-treated groups were analyzed by one-way analysis of variance (ANOVA) and Duncan’s multiple range tests (IBM SPSS Statistics 19, North Castle, NY, USA) with a significance threshold of *p* < 0.05.

## 3. Results

### 3.1. Hyperglycemic Rat Model

**Figure 1:**
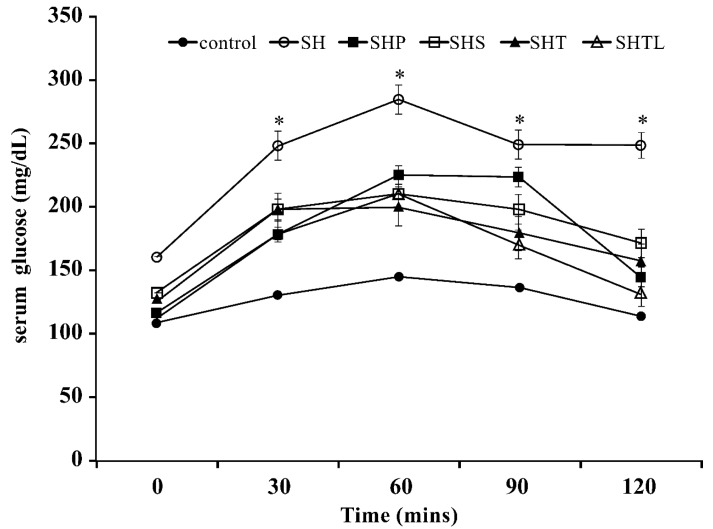
Oral glucose tolerance test (OGTT) for streptozotocin (STZ)-induced diabetic rats fed a high-fat diet for 14 weeks and administered *Lactobacillus plantarum* co-fermented tempeh orally during the last 4 weeks. Control: normal diet; SH: Streptozotocin (STZ 30 mg/kg, Nicotinamide 45 mg/kg) + High fat diet (Coconut oil 25%, Cholesterol 2%, Feed powder 73%); SHP: Streptozotocin (STZ 30 mg/kg, Nicotinamide 45 mg/kg) + High fat diet (Coconut oil 25%, Cholesterol 2%, Feed powder 73%) + Pioglitazone (10 mg/kg body weight); SHS: Streptozotocin (STZ 30 mg/kg, Nicotinamide 45 mg/kg) + High fat diet (Coconut oil 25%, Cholesterol 2%, Feed powder 73%) + Unfermented soybean (40 mg/kg body weight); SHT: Streptozotocin (STZ 30 mg/kg: Nicotinamide 45 mg/kg) + High fat diet (Coconut oil 25%, Cholesterol 2%, Feed powder 73%) + Tempeh (40 mg/kg body weight); SHTL: Streptozotocin (STZ 30 mg/kg, Nicotinamide 45 mg/kg) + High fat diet (Coconut oil 25%, Cholesterol 2%, Feed powder 73%) + Tempeh + *Lactobacillus plantarum* (40 mg/kg body weight). * Indicates a significant difference (*p* < 0.05) compared with the control group at the same time point. Results are expressed as mean values ± SD. (*n* = 8/group).

We induced DM in rats by STZ after 10 weeks of feeding on the HFD. The fasting serum glucose level was significantly higher (*p* < 0.05) in the STZ treatment group than in normal rats provided the chow diet (Figure 1). 

### 3.2. Oral Glucose Tolerance Test

In the treatment groups, serum glucose levels were ameliorated in DM rats after 14 weeks of HFD feeding (Figure 1). The serum glucose levels in the SH group (HFD) after the oral administration of glucose at 30, 60, 90, and 120 min were significantly higher than those of other treatment groups (*p* < 0.05). In addition, the OGTT showed that 40 mg/kg soybean (SHS group) and 40 mg/kg tempeh (SHT group) reduced the serum glucose level in STZ-induced DM rats. Moreover, the SHTL treatment group (40 mg/kg) exhibited significantly lower serum glucose levels than those in other treatment groups that are based on the OGTT (*p* < 0.05).

### 3.3. Effects of Various Treatments on Serum Biochemistry in DM Rats

In our serum biochemistry analysis, we observed significantly increased TG, cholesterol, LDL, FFA, serum glucose, HbA1C, and insulin levels, but reduced HDL levels in DM rats in the SH group after 14 weeks of the HFD (*p* < 0.05) (Table 1). The SH group achieved insulin resistance based on the HOMA-IR values. However, the SHS (40 mg/kg), SHT (40 mg/kg), and SHTL (40 mg/kg) treatments resulted in significant decreases in TG, cholesterol, LDL, FFA, serum glucose, HbA1C, and insulin levels, but increased HDL levels in DM rats (*p* < 0.05). In addition, the SHTL (40 mg/kg) treatment group exhibited the greatest improvements in all serum biochemical parameters, indicating that it could alleviate the symptoms of DM in rats; this group also exhibited improved insulin-resistance based on the HOMA-IR calculation.

### 3.4. Changes in Total Lactic Acid Bacteria in Diabetes Mellitus (DM) Rat Stools

There were no significant differences in the total LAB content in the rat stool samples before treatment among groups (Table 2). However, the total LAB content was lower in the SH group than in the Normal group. The total LAB contents were significantly higher in the SHT and SHTL groups than in the SH group in DM rats (*p* < 0.05). The total LAB content in the stool sample in the SHTL group was higher than those in other groups. However, the total LAB content in stool samples in the SHP group was significantly lower than those in all DM rats (*p* < 0.05).

### 3.5. Changes in Short Chain Fatty Acids (SCFAs) in DM Rat Stools

For STZ-induced DM rat groups within two weeks, there were no significant differences in acetic acid, propionic acid, and butyric acid in comparison with those in the SH group in DM rats (data not shown). However, the DM rats had higher SCFA contents than the rats fed a normal diet (Table 3). After four weeks of oral administration, the SHTL group exhibited significantly increased acetic acid, propionic acid, butyric acid, and valeric acid in stool samples compared with those in the SH group in DM rats (*p* < 0.05). The increases in acetic acid, propionic acid, and butyric acid in the SHTL group were the greatest when compared with those of other treatment groups.

### 3.6. Changes in Total Cholesterol, Bile Acid, and TG in DM Rat Stools

As shown in Table 4, there were no significant differences in stool weight, cholesterol (TC), bile acid, and TG before treatment among samples. However, the SH group had the lowest weights and excretion of TC and bile acid from the stool at 14 weeks among all the DM groups (*p* < 0.05). The SHT and SHTL groups exhibit greater bile acid contents than those of other groups in stool samples (*p* < 0.05), especially the SHTL group, which exhibited the highest bile acid excretion at 14 weeks in the DM rats (*p* < 0.05). The TG content in the SH group was significantly lower than those in the control, SHP, SHT, and SHTL groups. The excretion of TC, bile acid, and TG in the SHTL group was significantly higher than that in the SH group (*p* < 0.05). 

### 3.7. Microbiota Analysis of DM Rats

**Figure 2:**
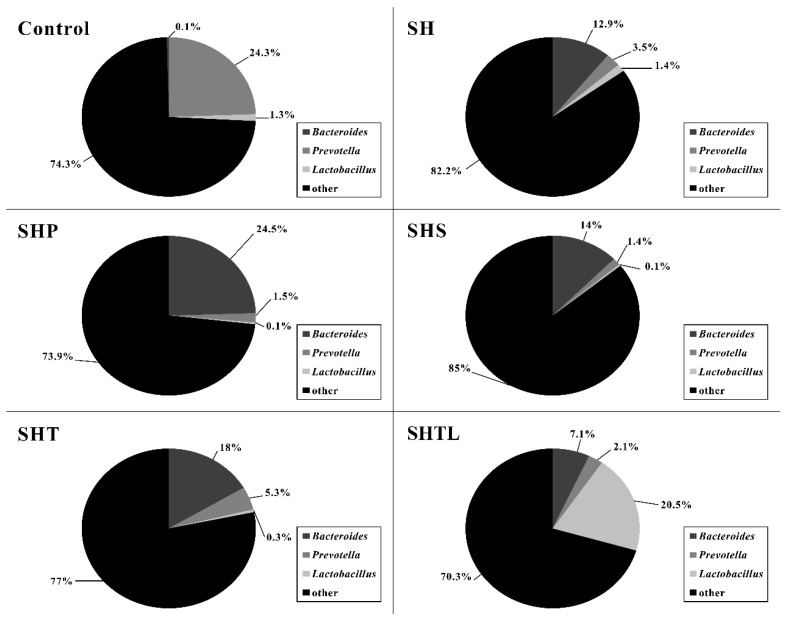
Changes in bacterial distribution in feces in STZ-induced diabetic rats fed a high-fat diet for 14 weeks and administered *Lactobacillus plantarum* co-fermented tempeh orally during the last 4 weeks. Control: normal diet; SH: Streptozotocin (STZ 30 mg/kg, Nicotinamide 45 mg/kg) + High fat diet (Coconut oil 25%, Cholesterol 2%, Feed powder 73%); SHP: Streptozotocin (STZ 30 mg/kg, Nicotinamide 45 mg/kg) + High fat diet (Coconut oil 25%, Cholesterol 2%, Feed powder 73%) + Pioglitazone (10 mg/kg body weight); SHS: Streptozotocin (STZ 30 mg/kg, Nicotinamide 45 mg/kg) + High fat diet (Coconut oil 25%, Cholesterol 2%, Feed powder 73%) + Unfermented soybean (40 mg/kg body weight); SHT: Streptozotocin (STZ 30 mg/kg: Nicotinamide 45 mg/kg) + High fat diet (Coconut oil 25%, Cholesterol 2%, Feed powder 73%) + Tempeh (40 mg/kg body weight); SHTL: Streptozotocin (STZ 30 mg/kg, Nicotinamide 45 mg/kg) + High fat diet (Coconut oil 25%, Cholesterol 2%, Feed powder 73%) + Tempeh + *Lactobacillus plantarum* (40 mg/kg body weight). Results are expressed as mean values ± SD. (*n* = 8/group).

We evaluated the distribution of gut bacteria by next-generation sequencing. The SH, SHP, SHS, and SHT groups exhibited a change in the dominant bacteria to Bacteroides in STZ-induced DM rats, and the second most dominant bacteria changed to Prevotella (Figure 2). Interestingly, in the SHTL group, the dominant bacteria in the stool samples was Lactobacillus (36.29%) after the oral administration of tempeh co-fermented with *L. plantarum* (40 mg/kg) in DM rats. The second most dominant bacterium in the SHTL group was Bacteroides (29.58%). The Lactobacillus content in the SHTL group was greater than that in the SH group by 34.2%.

## 4. Discussion

High serum glucose is a symptom of diabetes, and postprandial hyperglycemia is a metabolic phenomenon in type II diabetes [14,15]. Therefore, the objective of diabetes therapy is to control the fasting and postprandial serum glucose concentrations. Soybean isoflavones can be transformed from glycosides to aglycones by probiotics, and aglycone-isoflavones have better bioavailability in humans [16]. After treatment for four weeks, rats in each group were fasted for 12 h and then evaluated by OGTT (Figure 1). In our study, the SHTL group had better OGGT results in the late stage, and this was attributed to the high bioavailability of isoflavones from *L. plantarum* fermented with *Rhizopus oryzae* in tempeh in DM rats. Although the SHS and SHT groups had isoflavones, they exhibited decreased serum glucose in the OGTT. The higher serum glucose levels that were observed in the SHTL group than in other groups may reflect the higher aglycone-isoflavone content in the SHTL group. These results are consistent with previous findings [17].

The syndromes of insulin resistance are caused by abnormal responses of human tissues (such as the muscle, liver, adipocyte, and central nervous system tissues) to insulin, thereby inducing dysfunctions in glucose and lipid metabolism [18,19,20,21,22]. Insulin resistance normally co-exists with high blood pressure, hypertriglyceridemia, decreased HDL, increased LDL, and multiple metabolic disorder syndromes in animals. Hence, these syndromes could induce severe complications in patients with type II diabetes [23,24]. Animal and human studies consistently demonstrate that Lactobacillus can reduce the total cholesterol and LDL levels in the blood [25,26,27]. In addition, epidemiological and other studies have shown that isoflavonoids (genistein) in soybean could improve type II DM by regulating the metabolism of glucose and lipids [28,29]. Many studies have shown that isoflavonoids and daidzein of the soybean could reduce serum glucose levels in animals with DM [30,31,32]. As shown in Table 1, the SHLT co-fermentative group had better bioavailability, decreased TG and LDL levels, and increased HDL levels in the serum. Additionally, serum glucose and HbA1C levels were effectively regulated in the SHLT group. However, isoflavonoids (genistein) not only improved the metabolism of serum glucose, but also reduced the HOMA-IR value in DM rats. Our results were similar to those of Kwon (2010), who showed that fermented soybean can decrease TC and TG levels in the liver and can regulate the metabolism of serum glucose in SD rats [33].

Microbes that are beneficial to hosts are referred to as probiotics [34]. These probiotics, including LAB, need to survive in gastric acid and bile acid conditions in animals [35]. LAB can inhibit potential pathogen proliferation, decrease serum cholesterol levels, and regulate the immune system [36]. Furthermore, LAB in the stool can protect against gastric acid and bile acid damage. The consumption of soybean products also increases SCFAs, lactic acid bacteria, and the volume of stool [37]. Table 2 shows that total LAB increased significantly in soybean-fed groups. In particular, the SHLT group had the highest total LAB count in the stool. These findings are consistent with those of Panasevich [37].

Many studies have shown that increased dietary fiber intake can improve stool excretion, stimulate segmented colon movement, and improve blood sugar control [38,39,40,41,42,43]. Probiotics can produce active metabolites, such as SCFAs, in the gut. SCFAs are also a product of dietary fiber fermentation. They include acetate, propionate, and butyrate [44,45]. Some studies have shown that acetate is the most abundant SCFA in the serum and it can regulate inflammation and protect against the invasion of pathogens [46,47,48]. Propionate can decrease total cholesterol levels [49]. Butyrate can improve HFD-induced obesity and insulin sensitivity [50,51]. Table 3 demonstrates that the SHLT group exhibited increased acetate, propionate, and butyrate in the stool when compared with the levels in other groups. The results of Schneider (2006) supported our results for stool SCFAs [52].

Protein, isoflavones, or dietary fiber in soybeans would affect the metabolism of cholesterol [53,54,55]. LAB can improve the absorption of isoflavones by regulating β-galactosidase and glucosidase activity [56]. Glucosides of isoflavones are transformed to aglycone-isoflavones with better bioavailability via Lactobacillus [57]. In addition, increased consumption of aglycone-isoflavones improves fatty liver diseases [58]. Some results have demonstrated that the intake of soy products with dietary fiber can decrease serum total cholesterol and LDL-C levels, and the interaction of bile acid and microbes also regulates liver fat and the metabolism of cholesterol [59,60,61]. Recent studies have shown that the gut microbiota can affect intestinal-liver circulation and bile acid metabolism because it can produce new bile acid via decarboxylation, replacing the bile acid that is consumed by intestinal-liver circulation and decreasing the serum cholesterol level [62,63]. The consumption of dietary soy products can increase Lactobacillus spp. in the stool and promotes the activity of bile hydrolase [64]. Nagata (1982) also found that soy products could increase the bile acid content in rat feces and affects the metabolism of liver cholesterol, since bile acid synthesis requires cholesterol [65,66]. These results may be explained by the stimulation of bile acid secretion and the activity of 7α-hydroxylase cholesterol synthesis induced by LAB and isoflavones [67,68,69].

Prebiotics are a good source of probiotics and regulate cholesterol and blood sugar. They are typically derived from cereal fibers, such as β-dextran, arabinoxylan, inulin, galactose, and fructooligosaccharides [70,71]. Wang (2012) found that hemicellulose from cereals is composed of β-dextran, which can compete with cholesterol binding sites on LDL. Therefore, the consumption of dietary cereal fiber can decrease the serum levels of LDL and cholesterol [72,73]. Moreover, LAB can reduce blood cholesterol by various mechanisms, e.g., the inhibition of cholesterol synthesis enzymes, stimulation of cholesterol excretion in feces, and inhibition of cholesterol recycling, which can increase the synthesis of cholic acid [74]. Table 4 shows that the SHLT group exhibited dramatically increased levels of stool cholesterol and triglycerides. It is possible that LAB decreased cholesterol by each of these mechanisms, but it decreases blood cholesterol by increasing bile acid synthesis.

The gut microbiota is substantially influenced by the diet and it affects human health via microbial metabolism [75]. The gut microbiota in the human colon is also affected by the diet and induces metabolic diseases, like type II diabetes. In other words, dietary changes can improve physiological metabolism in humans by modifying microbial metabolic processes [76,77]. In a comparison of the gut microbiota, 53% of children in the African countryside, but not in Europe, had Prevotella. This may be explained by dietary differences since children in the African countryside consume cereal, soy, and vegetables and European children consume more protein and animal fat (and exhibit abundant Bacteroides in the gut) [78]. Prevotella and Bacteroides are major microbes in the human colon, and their distribution and metabolic activity are related to the diet. For example, Prevotella is abundant in those who eat a high fiber diet, but Bacteroides is abundant in those who eat high protein and high-fat diets [77]. Figure 2 also shows that the dominant bacteria in our HFD group were Bacteroides, but those in the normal control group were Prevotella.

Stool samples of children in the African countryside have four times higher levels of propionate and butyrate than those of samples from European children [78], and this difference might reflect the consumption of soy products, which increases Lactobacillus in stool samples [79]. Probiotics can increase SCFA production [79]. As shown in Figure 2, Lactobacillus was more abundant in the SHTL group than in other groups. Accordingly, the acetate, propionate, and butyrate contents were the highest in the stool samples of the SHTL group. These findings suggest that the SHTL group exhibits decreased serum glucose via increases in the proliferation of Lactobacillus and improvements in SCFA excretion.

## 5. Conclusions

The effects of *L. plantarum* co-incubated with *R. oligosporus* to produce soy tempeh on diabetes have not been evaluated. The present results demonstrate that *L. plantarum* co-incubation in soy tempeh ameliorates hyperglycemia, hyperlipidemia, and hyperinsulinemia by altering the intestinal bacterial distribution and increasing intestinal SCFA release in HFD-fed rats. These findings suggest that soy tempeh that is produced by co-incubation with *L. plantarum* has therapeutic effects and is a potential dietary supplement for preventing the progression of DM. 

## Figures and Tables

**Table 1:** Selected serum biochemical parameters for STZ-induced diabetic rats fed a high-fat diet for 14 weeks and administered *Lactobacillus plantarum* co-fermented tempeh orally during the last 4 weeks.

Items/Groups	Control	SH	SHP	SHS	SHT	SHTL
Triglyceride (mg/dL)	55.11 ± 20.0 ^bcd^	118.1 ± 35.8 ^a^	49.30 ± 8.52 ^cd^	71.50 ± 17.2 ^bc^	76.40 ± 24.7 ^b^	47.90 ± 9.95 ^d^
Cholesterol-total (mg/dL)	53.50 ± 6.86 ^c^	90.33 ± 11.1 ^a^	66.50 ± 13.4 ^bc^	79.67 ± 14.4 ^ab^	69.67 ± 14.4 ^bc^	65.50 ± 9.98 ^bc^
HDL-cholesterol (mg/dL)	40.56 ± 7.78 ^ab^	35.71 ± 4.59 ^b^	34.20 ± 6.16 ^b^	45.13 ± 10.3 ^a^	40.29 ± 4.08 ^ab^	40.14 ± 3.42 ^ab^
Cholesterol/HDL-C	1.41 ± 0.07 ^b^	2.12 ± 0.35 ^a^	2.04 ± 0.36 ^a^	2.02 ± 0.15 ^a^	1.94 ± 0.16 ^a^	2.01 ± 0.17 ^a^
LDL-cholesterol (mg/dL)	7.89 ± 2.23 ^c^	36.00 ± 8.68 ^a^	23.63 ± 7.20 ^b^	28.75 ± 9.77 ^b^	24.78 ± 6.29^b^	25.00 ± 5.24 ^b^
Free-fatty acid (mmol/L)	1.43 ± 0.61 ^b^	2.31 ± 0.25 ^a^	1.16 ± 0.06 ^b^	1.55 ± 0.23 ^b^	1.36 ± 0.31 ^b^	1.41 ± 0.24 ^b^
Glucose AC (mg/dL)	100 ± 8.4 ^c^	199 ± 42.3 ^a^	125 ± 34.6 ^bc^	151 ± 25.5 ^b^	141 ± 24.8 ^b^	109 ± 17.3 ^c^
HbA1C (%)	4.02 ± 0.13 ^d^	6.96 ± 1.05 ^a^	5.17 ± 0.97 ^bc^	5.58 ± 1.42 ^b^	5.51 ± 1.25 ^b^	4.42 ± 0.32 ^cd^
Insulin (ng/mL)	2.48 ± 2.11 ^b^	9.99 ± 5.46 ^a^	1.61 ± 0.81 ^b^	2.11 ± 0.67 ^b^	2.61 ± 0.53 ^b^	1.65 ± 0.53 ^b^
HOMA-IR	0.55 ± 0.18 ^c^	4.46 ± 0.95 ^a^	0.54 ± 0.19 ^c^	0.89 ± 0.17 ^bc^	1.07 ± 0.36 ^b^	0.59 ± 0.16 ^c^

Control: normal diet; SH: Streptozotocin (STZ 30 mg/kg, Nicotinamide 45 mg/kg) + High fat diet (Coconut oil 25%, Cholesterol 2%, Feed powder 73%); SHP: Streptozotocin (STZ 30 mg/kg, Nicotinamide 45 mg/kg) + High fat diet (Coconut oil 25%, Cholesterol 2%, Feed powder 73%) + Pioglitazone (10 mg/kg body weight); SHS: Streptozotocin (STZ 30 mg/kg, Nicotinamide 45 mg/kg) + High fat diet (Coconut oil 25%, Cholesterol 2%, Feed powder 73%) + Unfermented soybean (40 mg/kg body weight); SHT: Streptozotocin (STZ 30 mg/kg: Nicotinamide 45 mg/kg) + High fat diet (Coconut oil 25%, Cholesterol 2%, Feed powder 73%) + Tempeh (40 mg/kg body weight); SHTL: Streptozotocin (STZ 30 mg/kg, Nicotinamide 45 mg/kg) + High fat diet (Coconut oil 25%, Cholesterol 2%, Feed powder 73%) + Tempeh + *Lactobacillus plantarum* (40 mg/kg body weight). a~d letters are significantly different from all samples tested (*p* < 0.05). Results are expressed as mean values ± SD. (*n* = 8/group).

**Table 2:** Lactic acid bacteria counts (Log CFU/g) in STZ-induced diabetic rats in different treatment groups.

Items/Groups	Control	SH	SHP	SHS	SHT	SHTL
Week 0	7.66 ± 0.04 ^a^	7.65 ± 0.09 ^a^	7.64 ± 0.01 ^a^	7.59 ± 0.05 ^a^	7.75 ± 0.08 ^ab^	7.67 ± 0.05 ^a^
Week 4	8.91 ± 0.07 ^a^	8.09 ± 0.06 ^c^	7.71 ± 0.27 ^d^	8.04 ± 0.16 ^c^	8.31 ± 0.04 ^bc^	8.44 ± 0.05 ^b^

Control: normal diet; SH: Streptozotocin (STZ 30 mg/kg, Nicotinamide 45 mg/kg) + High fat diet (Coconut oil 25%, Cholesterol 2%, Feed powder 73%); SHP: Streptozotocin (STZ 30 mg/kg, Nicotinamide 45 mg/kg) + High fat diet (Coconut oil 25%, Cholesterol 2%, Feed powder 73%) + Pioglitazone (10 mg/kg body weight); SHS: Streptozotocin (STZ 30 mg/kg, Nicotinamide 45 mg/kg) + High fat diet (Coconut oil 25%, Cholesterol 2%, Feed powder 73%) + Unfermented soybean (40 mg/kg body weight); SHT: Streptozotocin (STZ 30 mg/kg: Nicotinamide 45 mg/kg) + High fat diet (Coconut oil 25%, Cholesterol 2%, Feed powder 73%) + Tempeh (40 mg/kg body weight); SHTL: Streptozotocin (STZ 30 mg/kg, Nicotinamide 45 mg/kg) + High fat diet (Coconut oil 25%, Cholesterol 2%, Feed powder 73%) + Tempeh + *Lactobacillus plantarum* (40 mg/kg body weight). a~d letters are significantly different from all samples tested (*p* < 0.05). Results are expressed as mean values ± SD. (*n* = 8/group).

**Table 3:** Changes in short- and medium-chain fatty acid in the feces in STZ-induced diabetic rats fed a high-fat diet for 14 weeks and administered *Lactobacillus plantarum* co-fermented tempeh orally during the last four weeks.

Week	Items	Groups					
		Control	SH	SHP	SHS	SHT	SHTL
**4**	**Acetic acid_C2**	4.16 ± 0.41 ^d^	5.21 ±0.11 ^c^	5.30 ± 0.29 ^c^	5.93 ± 0.31 ^c^	6.86 ± 0.28 ^b^	7.86 ±0.64 ^a^
	**Propanoic acid_C3**	0.55 ± 0.11 ^c^	0.70 ± 0.17 ^bc^	0.84 ± 0.19 ^abc^	1.01 ± 0.16 ^ab^	0.87 ± 0.07 ^ab^	1.13 ± 0.07 ^a^
	**Butyric acid_C4**	0.51 ± 0.06 ^abc^	0.27 ± 0.02 ^c^	0.45 ± 0.06 ^bc^	0.70 ± 0.21 ^ab^	0.57 ± 0.28 ^abc^	0.83 ± 0.11 ^a^
	**Isobutyic acid_C4t**	0.00 ± 0.01 ^a^	0.04 ± 0.05 ^a^	0.02 ± 0.01 ^a^	0.01 ± 0.02 ^a^	0.04 ± 0.02 ^a^	0.05 ± 0.01 ^a^
	**Valeric acid_C5**	0.03 ± 0.01 ^ab^	0.00 ± 0.00 ^b^	0.01 ± 0.02 ^b^	0.09 ± 0.07 ^ab^	0.05 ± 0.03 ^ab^	0.11 ± 0.04 ^a^
	**Isovaleric acid_C5t**	0.02 ± 0.01 ^a^	0.05 ± 0.06 ^a^	0.05 ± 0.01 ^a^	0.05 ± 0.01 ^a^	0.06 ± 0.03 ^a^	0.08 ± 0.02 ^a^
	**Caproic acid_C6**	0.00 ± 0.00 ^a^	0.00 ± 0.00 ^a^	0.00 ± 0.00 ^a^	0.00 ± 0.00 ^a^	0.01 ± 0.01 ^a^	0.01 ± 0.02 ^a^

Control: normal diet; SH: Streptozotocin (STZ 30 mg/kg, Nicotinamide 45 mg/kg) + High fat diet (Coconut oil 25%, Cholesterol 2%, Feed powder 73%); SHP: Streptozotocin (STZ 30 mg/kg, Nicotinamide 45 mg/kg) + High fat diet (Coconut oil 25%, Cholesterol 2%, Feed powder 73%) + Pioglitazone (10 mg/kg body weight); SHS: Streptozotocin (STZ 30 mg/kg, Nicotinamide 45 mg/kg) + High fat diet (Coconut oil 25%, Cholesterol 2%, Feed powder 73%) + Unfermented soybean (40 mg/kg body weight); SHT: Streptozotocin (STZ 30 mg/kg: Nicotinamide 45 mg/kg) + High fat diet (Coconut oil 25%, Cholesterol 2%, Feed powder 73%) + Tempeh (40 mg/kg body weight); SHTL: Streptozotocin (STZ 30 mg/kg, Nicotinamide 45 mg/kg) + High fat diet (Coconut oil 25%, Cholesterol 2%, Feed powder 73%) + Tempeh + *Lactobacillus plantarum* (40 mg/kg body weight). a~d letters are significantly different from all samples tested (*p* < 0.05). Results are expressed as mean values ± SD. (*n* = 8/group).

**Table 4:** Changes in weight, cholesterol, bile acid, and triglyceride contents in feces in STZ-induced diabetic rats fed a high-fat diet for 14 weeks and administered *Lactobacillus plantarum* co-fermented tempeh orally during the last 4 weeks.

Week	Items	Groups					
		Control	SH	SHP	SHS	SHT	SHTL
**Week 0**							
	**Feces weight (g)**	81.7 ± 1.75	81.0 ± 5.00	81.7 ± 3.06	80.7 ± 3.73	80.3 ± 4.30	81.7 ± 4.16
	**cholesterol content (mg/g)**	1.56 ± 0.31	1.47 ± 0.57	1.67 ± 0.16	1.66 ± 0.34	1.60 ± 0.26	1.43 ± 0.18
	**Bile acid content (µg/g)**	6.35 ± 0.51	6.22 ± 0.49	6.54 ± 0.41	6.84 ± 0.59	6.48 ± 0.35	6.56 ± 0.36
	**Triglyceride content (µg/g)**	57.52 ± 2.85	57.14 ± 3.48	58.12 ± 4.98	58.28 ± 2.78	57.04 ± 4.62	57.28 ± 2.14
**Week 4**							
	**Feces weight (g)**	81.7 ± 3.80 ^c^	79.7 ± 3.06 ^c^	83.0 ± 9.8 ^bc^	96.7 ± 4.16 ^ab^	100.0 ± 6.00 ^a^	104.0 ± 6.27 ^a^
	**cholesterol content (mg/g)**	4.90 ± 1.32 ^d^	27.5 ± 0.93 ^c^	29.2 ± 2.62 ^bc^	29.0 ± 3.13 ^bc^	32.1 ± 2.44 ^b^	35.6 ± 1.34 ^a^
	**Bile acid content (µg/g)**	4.63 ± 0.55 ^d^	176.4 ± 0.44 ^b^	247.7 ± 3.73 ^a^	115.9 ± 2.76 ^c^	173.0 ± 6.78 ^b^	248.2 ± 3.86 ^a^
	**Triglyceride content (µg/g)**	68.97 ± 1.76 ^a^	47.63 ± 3.45 ^c^	57.48 ± 2.01 ^b^	43.11 ± 0.24 ^c^	67.16 ± 3.15 ^a^	72.29 ± 8.87 ^a^

Control: normal diet; SH: Streptozotocin (STZ 30 mg/kg, Nicotinamide 45 mg/kg) + High fat diet (Coconut oil 25%, Cholesterol 2%, Feed powder 73%); SHP: Streptozotocin (STZ 30 mg/kg, Nicotinamide 45 mg/kg) + High fat diet (Coconut oil 25%, Cholesterol 2%, Feed powder 73%) + Pioglitazone (10 mg/kg body weight); SHS: Streptozotocin (STZ 30 mg/kg, Nicotinamide 45 mg/kg) + High fat diet (Coconut oil 25%, Cholesterol 2%, Feed powder 73%) + Unfermented soybean (40 mg/kg body weight); SHT: Streptozotocin (STZ 30 mg/kg: Nicotinamide 45 mg/kg) + High fat diet (Coconut oil 25%, Cholesterol 2%, Feed powder 73%) + Tempeh (40 mg/kg body weight); SHTL: Streptozotocin (STZ 30 mg/kg, Nicotinamide 45 mg/kg) + High fat diet (Coconut oil 25%, Cholesterol 2%, Feed powder 73%) + Tempeh + *Lactobacillus plantarum* (40 mg/kg body weight). a~d letters are significantly different from all samples tested (*p* < 0.05). Results are expressed as mean values ± SD. (*n* = 8/group).
